# Gut microbiome composition differences among breeds impact feed efficiency in swine

**DOI:** 10.1186/s40168-020-00888-9

**Published:** 2020-07-22

**Authors:** Matteo Bergamaschi, Francesco Tiezzi, Jeremy Howard, Yi Jian Huang, Kent A. Gray, Constantino Schillebeeckx, Nathan P. McNulty, Christian Maltecca

**Affiliations:** 1 Department of Animal Science, North Carolina State University, Raleigh, NC 27695 USA; 2 Matatu, Inc., 4340 Duncan Ave., Suite 211, St. Louis, MO 63110 USA; 3 Smithfield Premium Genetics, Rose Hill, NC 28458 USA

**Keywords:** Swine, Microbiome, Genetics, Performance, Feed efficiency

## Abstract

**Background:**

Feed efficiency is a crucial parameter in swine production, given both its economic and environmental impact. The gut microbiota plays an essential role in nutrient digestibility and is, therefore, likely to affect feed efficiency. This study aimed to characterize feed efficiency, fatness traits, and gut microbiome composition in three major breeds of domesticated swine and investigate a possible link between feed efficiency and gut microbiota composition.

**Results:**

Average daily feed intake (ADFI), average daily gain (ADG), feed conversion ratio (FCR), residual feed intake (RFI), backfat, loin depth, and intramuscular fat of 615 pigs belonging to the Duroc (DR), Landrace (LR), and Large White (LW) breeds were measured. Gut microbiota composition was characterized by 16S rRNA gene sequencing. Orthogonal contrasts between paternal line (DR) and maternal lines (LR+LW) and between the two maternal lines (LR versus LW) were performed. Average daily feed intake and ADG were statistically different with DR having lower ADFI and ADG compared to LR and LW. Landrace and LW had a similar ADG and RFI, with higher ADFI and FCR for LW. Alpha diversity was higher in the fecal microbial communities of LR pigs than in those of DR and LW pigs for all time points considered. Duroc communities had significantly higher proportional representation of the *Catenibacterium* and *Clostridium* genera compared to LR and LW, while LR pigs had significantly higher proportions of *Bacteroides* than LW for all time points considered. Amplicon sequence variants from multiple genera (including *Anaerovibrio*, *Bacteroides*, *Blautia*, *Clostridium*, *Dorea*, *Eubacterium*, *Faecalibacterium*, *Lactobacillus*, *Oscillibacter*, and *Ruminococcus*) were found to be significantly associated with feed efficiency, regardless of the time point considered.

**Conclusions:**

In this study, we characterized differences in the composition of the fecal microbiota of three commercially relevant breeds of swine, both over time and between breeds. Correlations between different microbiome compositions and feed efficiency were established. This suggests that the microbial community may contribute to shaping host productive parameters. Moreover, our study provides important insights into how the intestinal microbial community might influence host energy harvesting capacity. A deeper understanding of this process may allow us to modulate the gut microbiome in order to raise more efficient animals.

Video Abstract

## Background

Feed efficiency is an essential trait in pig production since feed accounts for 50 to 85% of pork producers’ costs [1, 2]. Feed conversion ratio and residual feed intake are two traits that have been routinely used to evaluate feed efficiency [3]. An animal with a low feed conversion ratio and low residual feed intake consumes less feed per unit of body weight than expected and is considered efficient, whereas an animal with a high feed conversion ratio and residual feed intake consumes more feed than expected and is considered inefficient [3]. Increasing a pig’s feed efficiency can thus decrease the total feed it consumes, reducing a farm’s costs and energy use [4]. Most efforts to optimize feed efficiency in pigs to date have focused on host genetics, management practices, and diet [5, 6]. Despite these factors that influence feed efficiency, little is known about the relationship between feed efficiency and different breeds of pigs. Do et al. (2013) [7] discovered breed differences in heritability estimates of feed efficiency as well as variations in their phenotypic and genetic correlations among Duroc, Landrace, and Large White.

The gut microbiota is a complex system that plays an important role in health and immunity in all mammals [8]. It is comprised of diverse populations of bacteria and other microorganisms whose abundances are impacted by both environmental and host genetic factors [9]. Recently, studies have demonstrated associations of microbial profiles with nutrition and productivity parameters [10–12]. Notably, the gut microbiota metabolizes various food components, providing nutrients to the host in the form of fermentation end-products and other by-products, amino acids, vitamins, and indole derivatives [13]. In the context of swine feed efficiency, the gut microbiota plays important roles in nutrient uptake, energy harvest, and carbohydrate metabolism, particularly in processing indigestible polysaccharides [14, 15]. Recent studies have reported that the composition and alpha diversity of the pig gut microbiota are correlated with nutrient digestibility, average daily gain, and body weight [11, 16]. Variation in the gut microbiome has also been associated with life stage [9, 17]. However, to the best of our knowledge, only a few studies have reported the effect of different microbial populations on feeding efficiency of different breeds. Singh et al. [18] reported a correlation between gut microbiota diversity and feed efficiency, while Tan et al. [19] identified differences in the microbiomes of pigs with high and low feed efficiencies. The genera *Bacteroides*, *Cellulosilyticum*, and *Prevotella,* were more abundant in low feed efficiency pigs, and *Oscillibacter* and *Rhodococcus* were found in animals that were more feed efficient [19, 20].

Duroc pigs are often used in breeding programs as the terminal sires in three-way crosses with Landrace × Large White or Large White × Landrace sows. The greater relative selection emphasis on feed efficiency and growth in Duroc compared with Landrace and Large White might have inadvertently selecting for more efficient microbiomes. Previous studies have reported gut microbiome differences when comparing hosts with different genetic backgrounds [21, 22]. This may partially explain why the association between the gut microbiome and feed efficiency shows low repeatability among studies [18–20]. In addition, diet has been reported to be a principal factor affecting gut microbiota composition [23]. The objectives of this study were (i) to characterize differences in feed efficiency, growth, and fatness traits between three different commercial breeds of pigs; (ii) to study how the composition of the gut microbiome within and between breeds changes as animals grow; and (iii) to investigate whether there is an association between gut microbiome composition and feed efficiency in swine.

## Results

Duroc pigs are currently used in breeding programs as paternal lines, while crosses between Landrace and Large White pigs are used as maternal lines. The different aims of selection for paternal and maternal lines might have significantly shaped feed efficiency and gut microbiome composition. For this reason, to investigate the differences between paternal and maternal lines and between the two maternal lines with the minimum number of orthogonal contrasts, we performed the comparison between Duroc and the average of Landrace and Large White [DR vs. (LR and LW)/2] and Landrace versus Large White (LR vs. LW). In this study, the “Results” section was divided into four parts. In the first part, we reported differences between breeds in terms of feed efficiency and fatness traits. In the second part, gut microbiome differences between breeds at each time point were showed. In the third part, the effects of amplicon sequence variants (ASV) and the interaction between ASV and breed on feed efficiency were reported separately at the three time points. Lastly, the associations between gut microbiome and feed efficiency were presented.

### Feed efficiency and fatness traits in the Duroc, Landrace, and Large White breeds

Table 1 and Additional file 1 summarizes descriptive statistics and breed difference estimates for average daily feed intake (ADFI), average daily gain (ADG), feed efficiency, and fatness traits. Average daily feed intake, ADG, back fat, and loin depth were all lower in Duroc (DR) pigs than the average of Landrace (LR) and Large White (LW) animals. Landrace and LW pigs were similar in terms of ADG, residuals calculated by regressing ADFI on ADG (RF1), residuals calculated by regressing ADFI on ADG and body weight (RF2), and intramuscular fat (IMF); however, LW pigs had significantly higher ADFI, feed conversion ratio (FCR), and backfat.

**Table 1:** Effect of breed on growth, feed efficiency, and fatness traits

	Duroc (DR)		Landrace (LR)		Large White (LW)		Contrasts (*p* value)		RMSE
	LSM	SE	LSM	SE	LSM	SE	DR vs. (LR+LW)/2	LR vs. LW	
Efficiency									
ADFI, g/d	2185.1	51.27	2213.9	51.9	2396.8	51.7	0.061	0.015	267.4
ADG, g/d	618.4	10.9	627.1	11.1	649.91	11.0	0.142	0.149	56.8
RF1, g	− 4.61	51.38	− 4.90	52.30	21.17	52.50	0.842	0.727	230.3
RF2, g	− 1.00	45.45	− 9.59	46.14	14.84	46.28	0.949	0.710	219.6
FCR	3.56	0.08	3.54	0.08	3.70	0.08	0.530	0.154	0.38
Fatness traits									
Back fat, mm	10.33	0.34	11.43	0.34	13.93	0.29	< 0.001	< 0.001	2.36
Loin depth, mm	49.60	0.54	47.97	0.54	46.37	0.50	< 0.001	0.033	4.44
IMF, %	2.04	0.07	1.80	0.07	1.96	0.06	0.065	0.086	0.68

*LSM* least squares mean, *SE* standard error, *RMSE* root mean square error, *ADFI* average daily feed intake, *ADG* average daily gain, *RF1* residuals calculated regressing ADFI on ADG, *RF2* residuals calculated regressing ADFI on ADG and body weight, *FCR* average feed conversion ratio calculated as the ratio between ADFI and ADG, *IMF* intramuscular fat

**Fig. 1:**
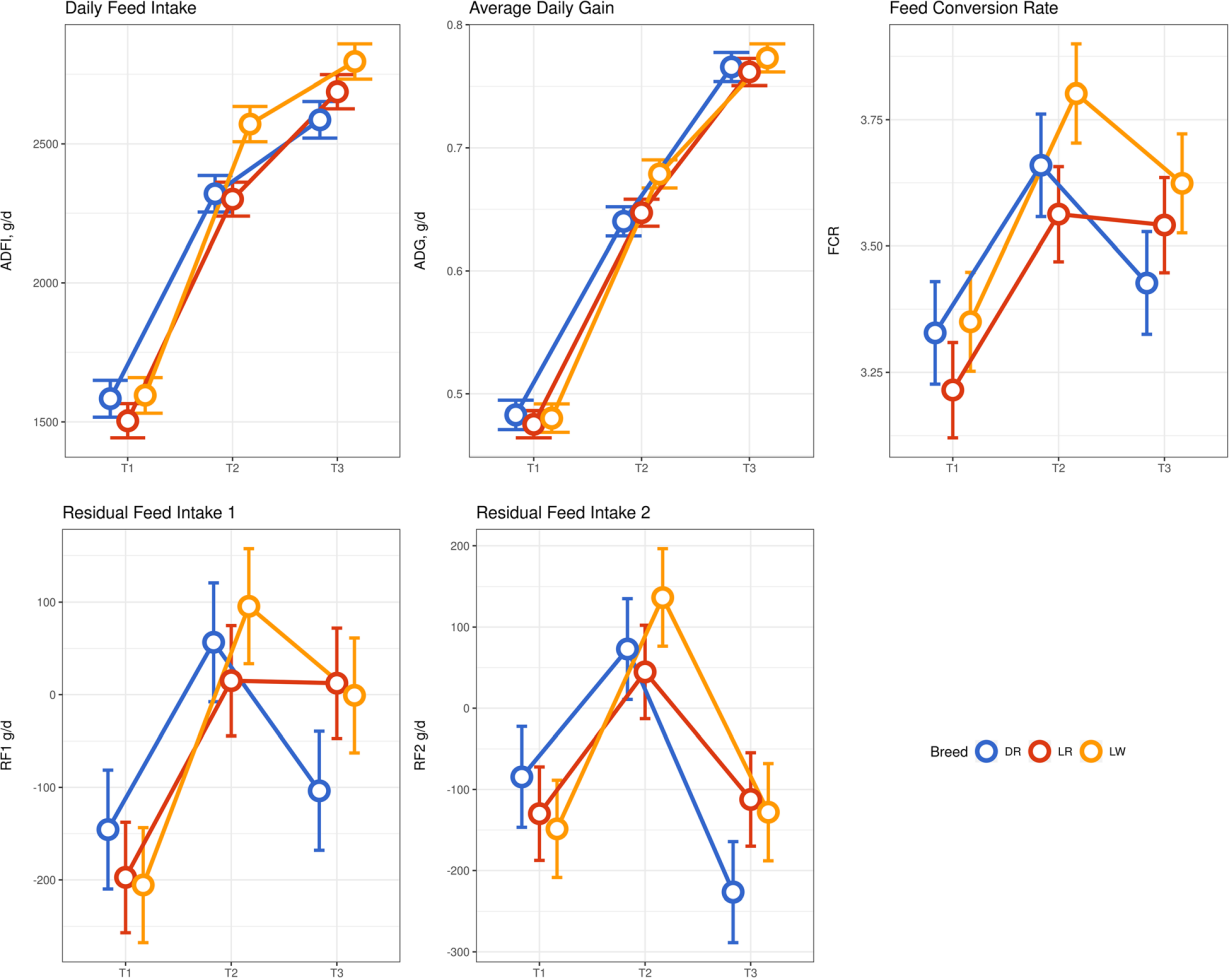
Average daily gain and feed efficiency (least squares mean ± confidence interval) of Duroc (DR), Landrace (LR), and Large White (LW) at three time points 73 days (T1), 123 days (T2), and 158 days (T3) in the growth trial

**Fig. 2:**
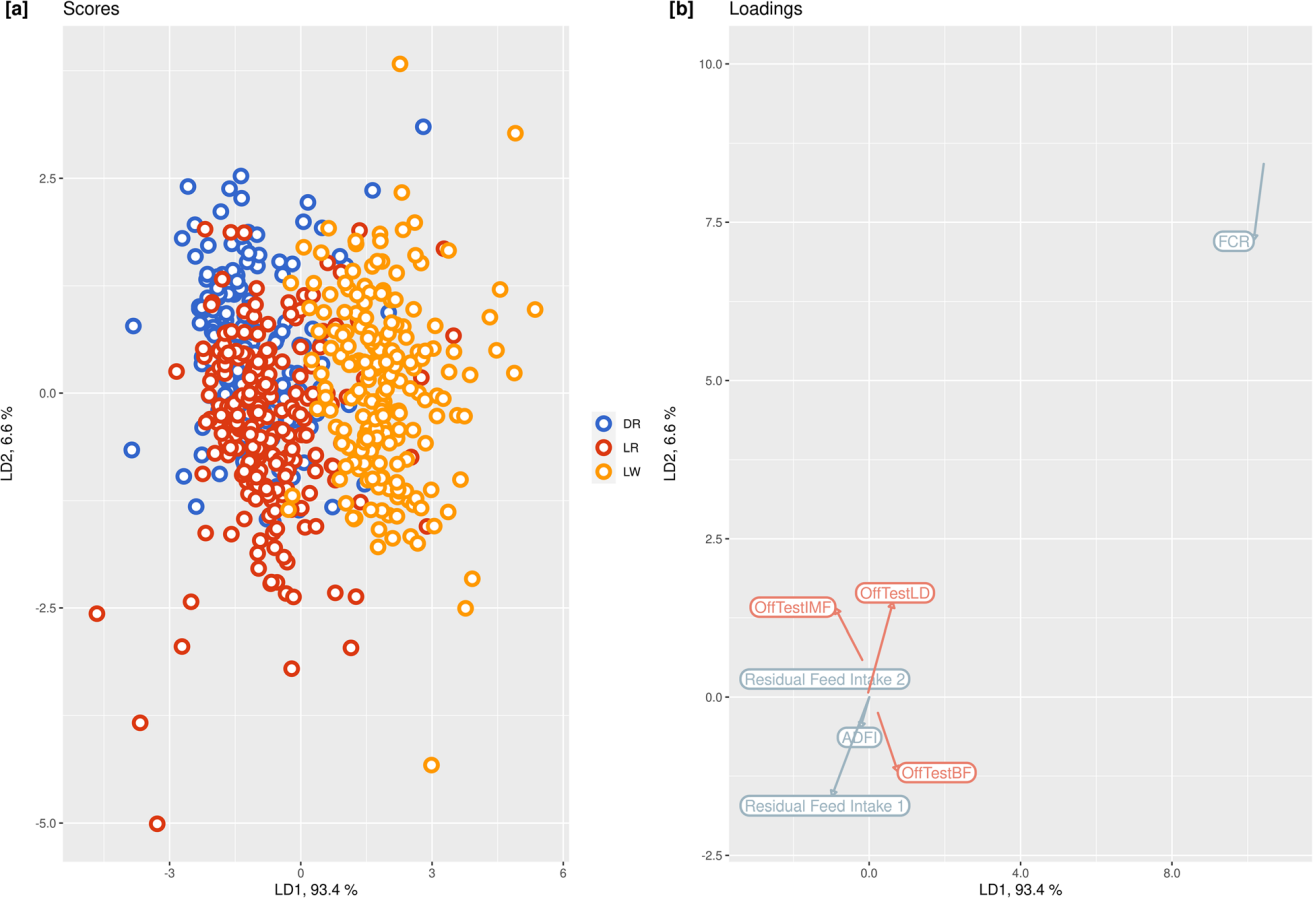
Linear discriminant analysis (LDA) of feed efficiency and fatness trait data from three breeds of pig. Scores [**a**] and loadings [**b**] are reported

Least squares means for ADFI, ADG, feed conversion ratio, residual feed intake 1 (RF1), and residual feed intake 2 (RF2) in each of the three breeds across three time points (73, 123, and 158 days) are reported in Fig. 1. Significant (*P* < 0.05) differences were observed. Across time points, ADG for the three breeds rang

ed from 0.48 to 0.77 kg/d. At 123 days, ADG was significantly higher in LW (0.68 ± 0.058 kg/d) than DR (0.64 ± 0.057 kg/d) and LR (0.65 ± 0.055 kg/d), while no statistically significant differences were observed at 73 and 158 days. Average daily feed intake ranged from 1503 to 2795 g/d across time points. Average daily feed intake was significantly higher in LW at 123 and 158 days than in DR and LR, while no significant differences were observed at 73 days. The FCR of the three breeds on average ranged from 3.21 to 3.80 across time points. Feed conversion ratio was significantly lower in LR at 73 and 123 days compared to DR and LW. With regard to the residual feed intake, RF1 ranged from − 205 to 95, while RF2 ranged from − 226 to 136 across time points. These significant differences between breeds were confirmed by linear discriminant analysis (LDA) (Fig. 2). The first component explained 93.4% and the second 6.6% of the total variance. The first component differentiated DR and LR from LW based on FCR (loading 10.4) and IMF (loading − 0.18), while the second component discriminated DR and LR based on FCR (loading 8.4) and backfat (loading − 0.25).

### Gut microbiome composition in the Duroc, Landrace, and Large White breeds

**Fig. 3:**
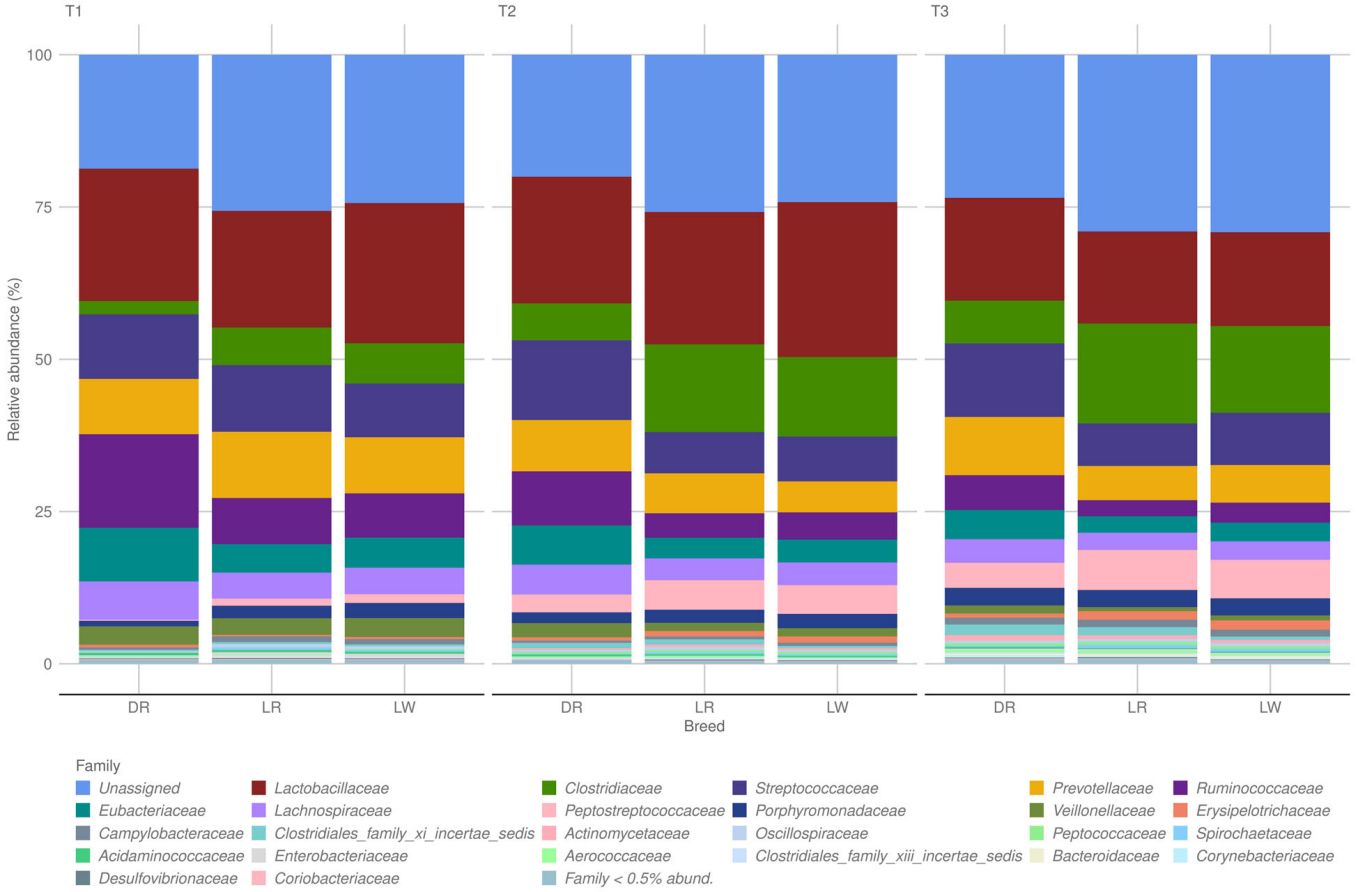
Relative abundance of microbiome taxa at family level of Duroc (DR), Landrace (LR), and Large White (LW) in three time points 73 days (T1), 123 days (T2), and 158 days (T3) of the feeding trial

Figure 3 illustrates the relative abundance of microbial ASV when aggregated at the family level for the three breeds and the three time points surveyed in this study. Over the three time points, about 80% of ASV were classified into just 7 families: *Lactobacillaceae*, *Clostridiaceae*, *Streptococcaceae*, *Prevotellaceae*, *Ruminococcaceae*, *Eubacteriaceae*, and *Lachnospiraceae.*

**Fig. 4:**
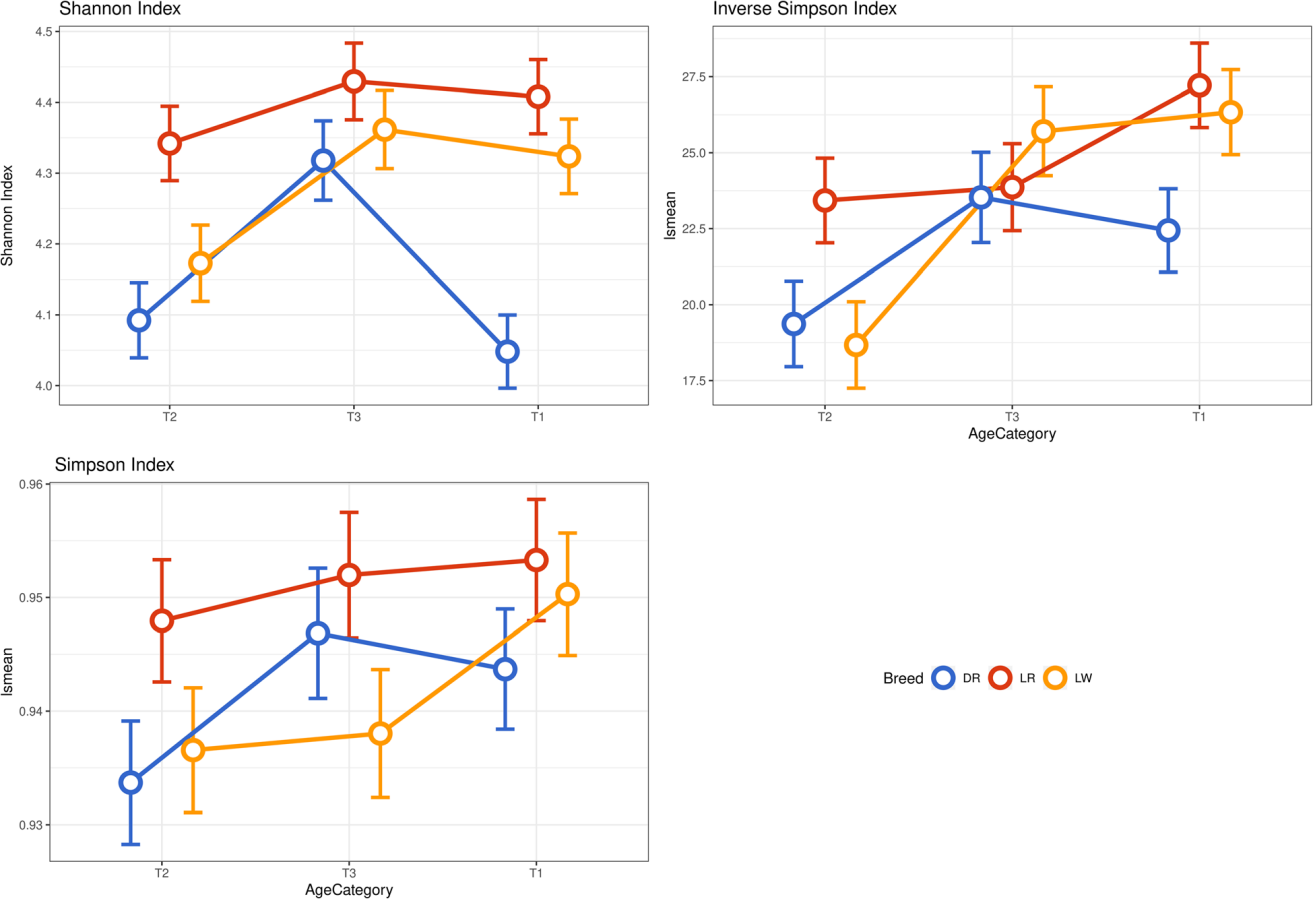
Measurements of fecal microbiome alpha diversity at ASV level using the Shannon, Simpson, and Inverse Simpson indices (least squares means ± confidence interval) in the Duroc (DR), Landrace (LR), and Large White (LW) breeds for three time points 73 days (T1), 123 days (T2), and 158 days (T3) in the growth trial.

Alpha diversity of pig gut microbiome was measured using the Shannon, Simpson, and Inverse Simpson indices (Fig. 4). On average, the alpha diversity across time points ranged from 4.05 to 4.43, from 0.93 to 0.95, and from 18.7 to 27.2 for the Shannon, Simpson, and Inverse Simpson indices, respectively. Comparing the breeds across time points, Shannon index values were significantly (*P* < 0.05) higher in LR than in DR and LW at 123 days. Duroc pigs had lower Shannon index values than LR and LW at 73 days, while no statistically differences were observed at 123 and 158 days. Simpson index values were lower in DR than in LR and LW at 73 and 123 days. The Inverse Simpson index was significantly lower in DR than in LR and LW at 73 days. Landrace had a higher Inverse Simpson index than DR and LW at 123 days, while no statistically significant differences were observed at 158 days.

**Fig. 5:**
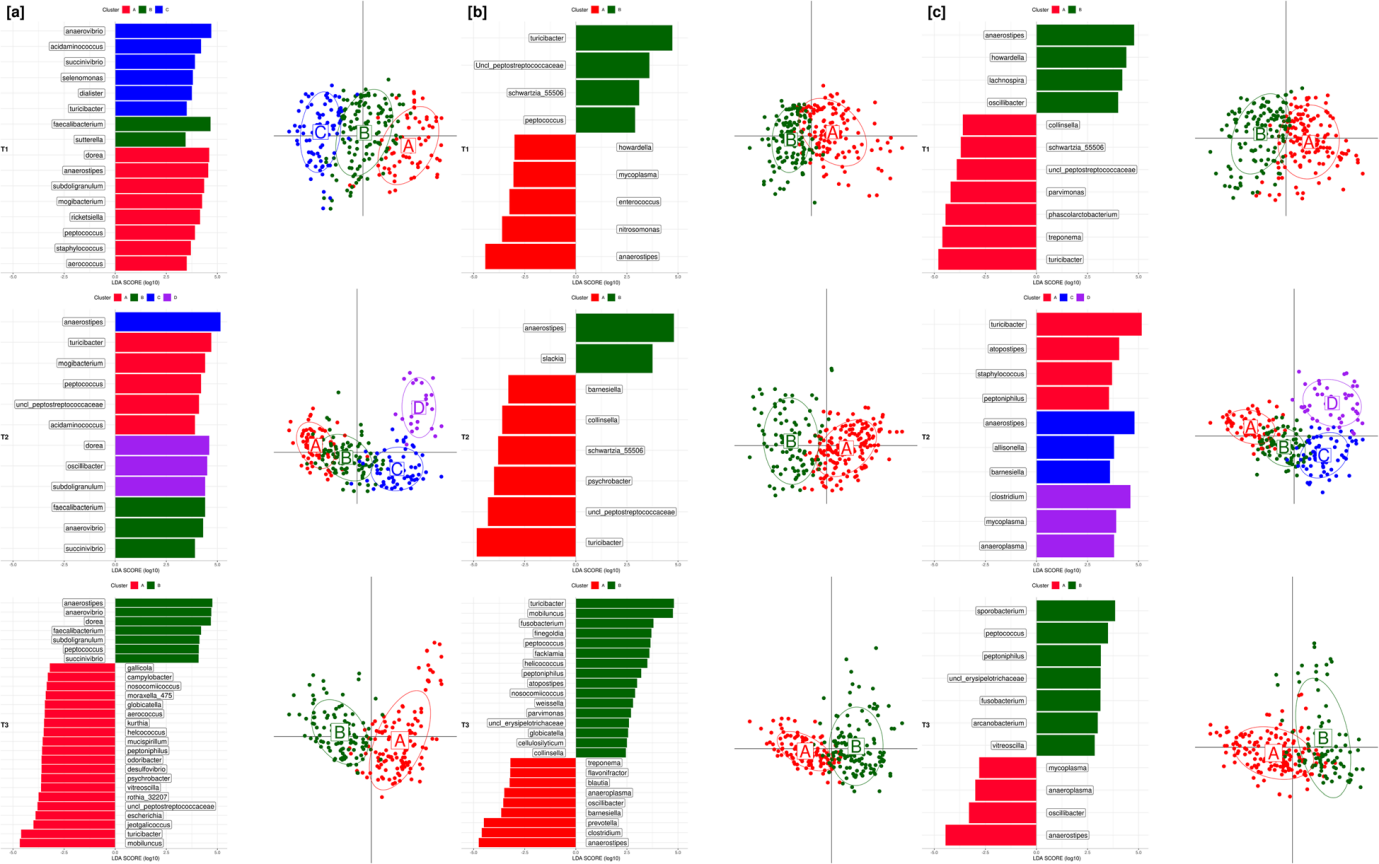
Clustering and LDA analyses of gut microbiome data collected from Duroc [**a**], Landrace [**b**], and Large White [**c**] pigs at three time points 73 days (T1), 123 days (T2), and 158 days (T3) of the feeding trial. (Above) Genera that differentiate enterotypes at each time point. Bar length represents a log_10_-transformed linear discriminant score. Color corresponds to the enterotype in which a genus was found to be most abundant. (Below) Enterotype clusters identified using Calinski–Harabasz (CH) index maximization

Clustering analyses were focused on identifying clusters (enterotypes) among the fecal samples of pigs collected at each time point. Each of these enterotypes may be driven by specific genera that contribute to microbial compositions. The clusters of samples and genera that significantly separate the enterotypes according to breed and time points are shown in Fig. 5 and Supplementary Figure S1. The optimal number of clusters based on Calinski–Harabasz index maximization was 2, with the exception of 73 and 123 days for DR and LW samples where the optimal number of clusters was more than 2. In order to identify specific bacterial genera that were characteristic to the three breeds within each time point, we performed an LDA analysis coupled with LDA Effect Size LEfSe. Figure 5 shows the genera that were differentially represented among the three breeds and time points. *Anaerostipes* and *Turicibacter* genera had very high LDA scores across all breeds and enterotypes. At 73 days, the three enterotypes in DR pigs were distinguished by *Dorea*, *Faecalibacterium*, and *Anaerovibrio*, while the enterotypes for LR and LW pigs were distinguished by *Anaerostipes* and *Turicibacter*. At 123 days, the four enterotypes in DR pigs were distinguished by a total of 12 genera. Of these, *Turicibacter*, *Faecalibacterium*, *Anaerostipes*, and *Dorea* were most abundant in enterotypes A, B, C, and D, respectively. At the same time point, the two enterotypes for LR were significantly (*P* < 0.05) distinguished for either *Turicibacter* or *Anaerostipes*, while the three enterotypes for LW were significantly (*P* < 0.05) distinguished by *Turicibacter*, *Anaerostipes*, and *Clostridium*. Enterotypes A and B for DR and LR at 158 days were significantly (*P* < 0.05) distinguished by *Turicibacter* and *Anaerostipes*, while the two enterotypes for LW were distinguished by 11 genera. Of these, *Anaerostipes* and *Sporobacterium* were significantly (*P* < 0.05) more abundant. This analysis revealed a different grouping of the samples across time points for DR and LW, while the LR samples were mainly clustered into *Anaerostipes* and *Turicibacter* enterotypes.

### ASV differentially represented in the Duroc, Landrace, and Large White breeds

Analyses of ASV representation performed at each time point revealed the gut microbiomes of DR, LR, and LW pigs to be distinct (Supplementary Table S1). When ASV abundances in DR pigs were compared to the average of those in LR and LW pigs at 73, 123, and 158 days, a total of 441, 401, and 324 ASV were found to be significantly different in terms of their representation (false discovery rate (FDR), 5%). Of these, 261 ASV classified as *Firmicutes* (177), *Bacteroidetes* (55), *Proteobacteria* (12), *Spirochaetes* (11), *Actinobacteria* (3), *Chlamydiae* (1), *Fusobacteria* (1), and *Tenericutes* (1) were shared between 73 and 123 days; 267 ASV classified as *Firmicutes* (180), *Bacteroidetes* (59), *Proteobacteria* (11), *Spirochaetes* (10), *Actinobacteria* (5), *Fusobacteria* (1), and *Tenericutes* (1) were shared between 123 and 158 days, and 191 ASV classified as phylum *Firmicutes* (128), *Bacteroidetes* (41), *Proteobacteria* (9), *Spirochaetes* (8), *Actinobacteria* (3), *Fusobacteria* (1), and *Tenericutes* (1) were shared between 73 and 158 days.

The relative abundances of 184, 153, and 123 ASV were significantly different between LR versus LW pigs at 73, 123, and 158 days, respectively. Of these, 56 consistently separated LR from LW pigs at 73 and 123 days, specifically, *Firmicutes* (24), *Bacteroidetes* (16), *Proteobacteria* (9), *Spirochaetes* (3), *Actinobacteria* (3), and *Fusobacteria* (1). There were 38 ASV that were systematically different between LR and LW at 123 and 158 days. These belonged to different phyla such as *Firmicutes* (24), *Bacteroidetes* (5), *Proteobacteria* (5), *Actinobacteria* (3), and *Spirochaetes* (1). Thirteen ASV classified as *Firmicutes* (5), *Bacteroidetes* (4), *Proteobacteria* (3), and *Actinobacteria* (1) were shared between 73 and 158 days.

**Fig. 6:**
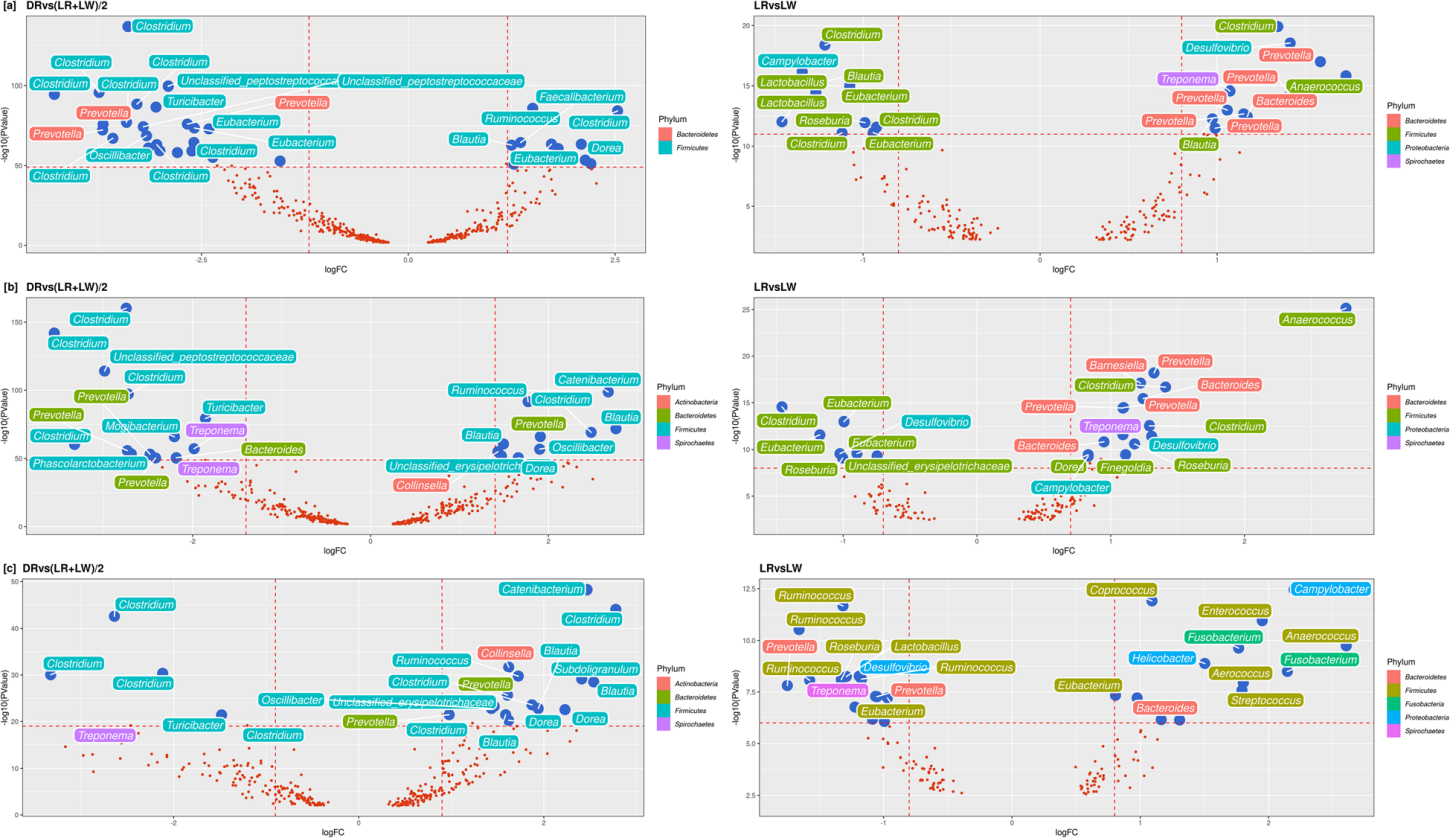
ASV with significantly higher or lower relative abundance when comparing the Duroc (DR) sire line vs the average of the Landrace and Large White maternal lines (DR vs (LR+LW)/2) (left panel) or the two maternal lines (LR vs LW) (right panel) at three time points (73 days (T1) [**a**], 123 days (T2) [**b**], and 158 days (T3) [**c**]). Each volcano plot simultaneously indicates the log_2_ fold change (*x*-axis) and the –log_10_(*p* value) (*y*-axis) for each ASV plotted. Dashed lines indicate arbitrary thresholds of logFC and *p* value. Each point represents an ASV, annotation highlight the genus of the ASV, while color represents the phylum.

The significant ASV in the orthogonal contrasts of DR versus the average of LR and LW and LR versus LW at all three time points are presented as volcano plots (Fig. 6a–c), while the relative abundance is reported in Additional file 2. A comparison of the Duroc sire line with the combined LR and LW maternal lines at 73 days revealed a higher abundance of 6 *Firmicutes*. The most significant of these were classified as *Ruminococcus* and *Clostridium.* In a comparison of the two maternal lines with one another at 73 days, LR had higher representation of *Bacteroidetes* and *Spirochaetes* than LW. Within these phyla, the most significant genera were *Prevotella*, *Bacteroides*, and *Treponema*. Landrace also had a lower proportion of several ASV than LW at 73 days, many of which were classified as *Clostridium*, *Campylobacter*, *Blautia, Eubacterium*, *Lactobacillus*, and *Roseburia* (Fig. 6a).

Significant differences were also observed at 123 days when comparing ASV abundances in DR pigs with the average of LR and LW. Duroc pigs had higher proportion of 8 *Firmicutes*, 1 *Actinobacteria*, and 1 *Bacteroidetes* than the two maternal lines. The most abundant genera within these phyla were *Catenibacterium*, *Prevotella*, and *Collinsella*. On the other hand, DR, compared to the average of LR and LW at 123 days had a lower proportion of 8 *Firmicutes*, 4 *Bacteroidetes*, and 2 *Spirochaetes.* Within these phyla, the most significant genera were classified as *Clostridium*, *Prevotella*, and *Treponema*. Remarkable differences in ASV abundances were also observed when comparing LR to LW at 123 days. Specifically, LR had a higher proportion of 6 *Bacteroidetes*, 6 *Firmicutes*, 2 *Proteobacteria*, and 1 *Spirochaetes*. Within these phyla, the predominant genera were *Anaerococcus*, *Barnesiella*, *Clostridium*, and *Prevotella.* Interestingly, the gut microbiome of LR at 123 days also had a lower proportion of ASV belonging to *Clostridium* and *Eubacterium* than LW (Fig. 6b).

The orthogonal contrast between paternal and maternal lines at 158 days revealed a higher abundance of 13 *Firmicutes*, 2 *Bacteroidetes*, and 1 *Actinobacteria*. Within the *Firmicutes* phylum, as observed in 73 and 123 days, the most significant genera were *Catenibacterium* and *Clostridium.* The paternal line had a lower abundance of 5 *Firmicutes* and 1 *Spirochaetes*. Of these, one of the most significant genera was *Turicibacter* as observed in 73 and 123 days. We also discovered differences between the two maternal lines at 158 days. LR compared to LW had a particularly higher abundance of *Bacteroides*, *Campylobacter*, *Coprococcus*, *Enterococcus*, and *Fusobacterium*, as well as a lower abundance of *Lactobacillus*, *Ruminococcus*, and *Desulfovibrio* (Fig. 6c). Of these, the genus *Bacteroides* was the most significant discriminant of LR and LW across the three time points.

### Association between pig phenotypes and gut microbiota

We identified several ASV whose relative abundances correlated significantly with feed efficiency and fatness traits (Supplementary Table S2). A total of 16, 33, and 93 ASV were significantly associated with feed efficiency and fatness traits at 73, 123, and 158 days, respectively. They belonged mainly to 4 phyla: *Firmicutes*, *Bacteroidetes*, *Proteobacteria*, and *Spirochaetes*. At the genus level, 14 were classified as *Ruminococcus*, 12 as *Clostridium*, 10 as *Eubacterium*, 6 as *Lactobacillus*, 5 as *Bacteroides*, and 4 as *Prevotella*. Twelve ASV were classified as belonging to 7 other minor genera, and 79 were unassigned. We identified 20, 1, 20, 26, 61, 6, and 8 taxa that were significantly associated with ADFI, ADG, RF1, RF2, FCR, backfat, and loin depth, respectively, at the three different time points.

The interaction effects between ASV and breed were significant for 2 and 48 ASV at 73 and 158 days (Supplementary Table S3), respectively, while no significant interaction effects were observed at 123 days. The significant ASV belonged to the *Firmicutes* and *Bacteroidetes*. Eight of these belonged to the *Faecalibacterium* genus, 4 to the *Eubacterium*, 4 to the *Bacteroides*, 4 to the *Oscillibacter*, 4 to the *Ruminococcus*, 2 to the *Anaerovibrio*, and 24 were unassigned. Of the ASV identified, 22, 22, and 4 were significantly associated with ADFI, RF1, and FCR, respectively, at 73 and 158 days.

We found across time points that the genus *Peptococcus* and *Turicibacter* were positively correlated (*r*_s_ ~ 0.27) with backfat at 123 and 158 days. The genera *Faecalibacterium* and *Oscillibacter* were negatively correlated with feed efficiency (*r*_s_ = − 0.22) at 73 days. A negative correlation (*r*_s_ ~ − 0.20) was also found between *Anaerovibrio*, *Catenibacterium*, *Dorea*, and *Roseburia* and fatness traits at 73, 123, and 158 days (Additional file 3).

**Fig. 7:**
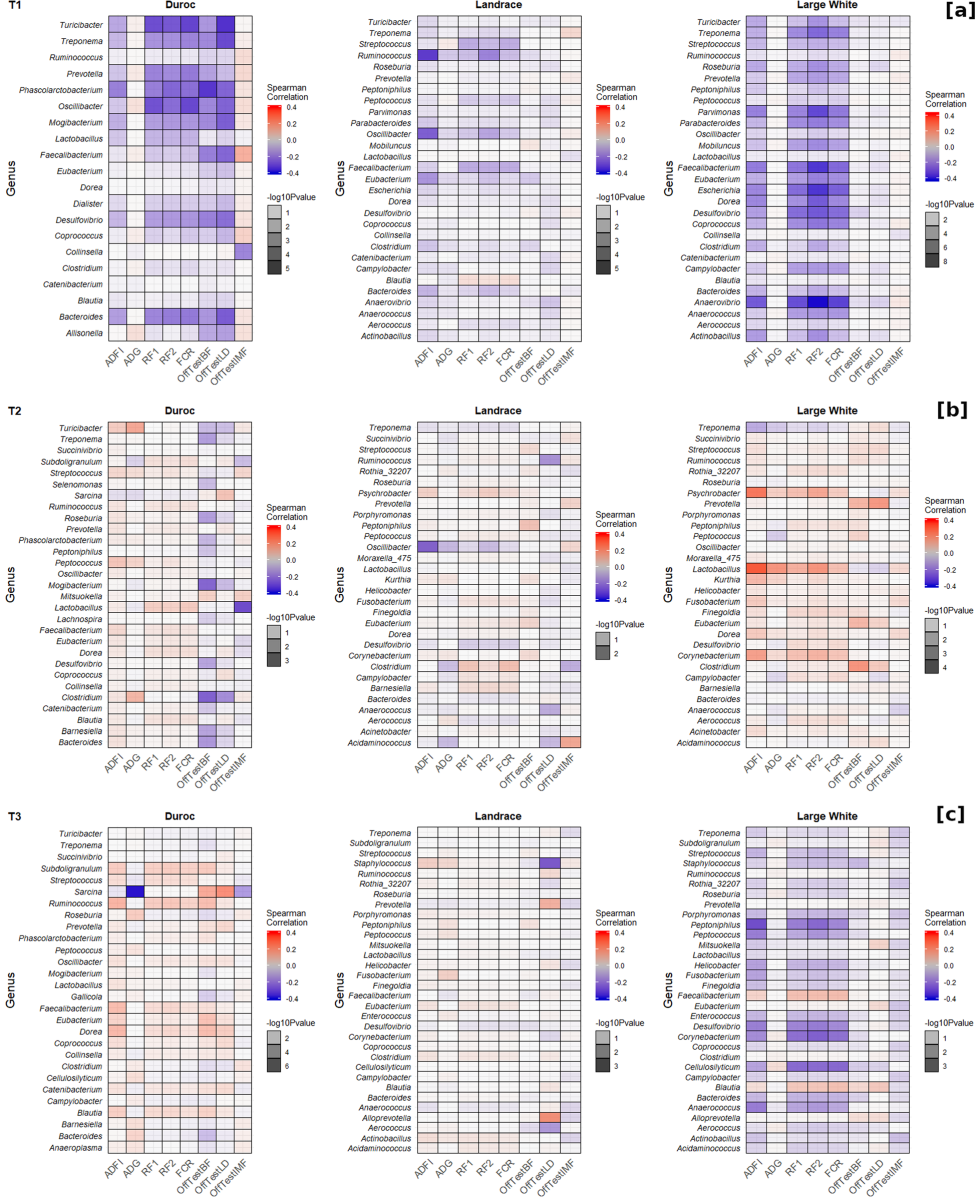
Heat maps showing Spearman correlations and level of significance between genera, feed efficiency, and fatness traits of Duroc, Landrace, and Large White in three time points of the growth trial (73 days (T1) [**a**], 123 days (T2) [**b**], and 158 days (T3) [**c**]). Correlations were analyzed between ASV at the genus level. The color represents the correlation while the saturation represents the level of significance

We discovered within breed and across time points that the genus *Oscillibacter* was negatively correlated (*r*_s_ ~ − 0.30) with feed efficiency (RF1, RF2, and FCR), backfat, and loin depth in DR at 73 days. The genera *Blautia*, *Dorea*, *Eubacterium*, *Faecalibacterium*, *Lactobacillus*, and *Ruminococcus*, on the other hand, were positively correlated with feed efficiency (*r*_s_ ~ 0.15) and fatness traits (*r*_s_ ~ 0.22) in Duroc pigs at 123 and 158 days. Similarly, a negative correlation was obtained between the genus *Sarcina* and growth (*r*_s_ = − 0.38) as well as IMF (*r*_s_ = − 0.23) at 158 days (Fig. 7a–c).

The genus *Oscillibacter* was negatively correlated (*r*_s_ ~ − 0.17) with ADFI and feed efficiency in LR at 73 and 123 days. This maternal line also had a positive correlation (*r*_s_ ~ 0.17) at 123 and 158 days between ASV classified as genera *Clostridium* and feed efficiency.

In addition, we found a positive correlation (*r*_s_ ~ 0.20) between four genera (*Corynebacterium*, *Lactobacillus*, *Finegoldia*, and *Psychrobacter*) and both ADFI and feed efficiency in LW pigs at 123 days, while the genus *Desulfovibrio* was negatively correlated (*r*_s_ ~ − 0.33) with ADFI, feed efficiency, and backfat at 73 and 158 days.

We found within time points and across breeds that the genus *Oscillibacter* was negatively correlated with ADFI and feed efficiency for DR, LR, and LW pigs at 73 days. The genera *Anaerovibrio*, *Clostridium*, *Faecalibacterium*, *Eubacterium*, and *Ruminococcus,* showed negative correlations with feed efficiency and backfat in both LR and LW at 73 days. ASV classified as *Dorea*, *Eubacterium*, and *Lactobacillus* were positively correlated with feed efficiency in DR and LW at 123 days. Additionally, the genus *Blautia* was positively correlated with feed efficiency in DR and LR at 123 days. ASVs classified as *Dorea* and *Lactobacillus* were positively correlated with ADFI and feed efficiency in DR and LW at 158 days.

## Discussion

In the past decade, many studies have investigated the potential impact of the swine microbiome on different phenotypes [9, 11]. However, few studies have compared the gut microbiomes of different pig breeds [21, 24]. To the best of our knowledge, this is the first large-scale study that explores the relationship between breed, gut microbiome composition, and feed efficiency. The data used in this study were collected from DR, LR, and LW, which are the most common commercial breeds worldwide due to their favorable lean growth, maternal behavior, feed efficiency, and production traits [25, 26].

The gut microbiome of all three breeds was dominated by the phyla *Firmicutes* and *Bacteroidetes*, consistent with previous findings [9, 19, 27]. However, the most abundant bacterial family in the breeds characterized here was *Lactobacillaceae*, rather than *Clostridiaceae* and *Prevotellaceae*, as reported by Lu et al. [9]. Several factors potentially contributed to gut microbiota variation, among them breed, age, body weight, and diet [17]. Furthermore, previous studies revealed a significant effect of host genetics and environmental factors (e.g., pen, kinship) on gut microbiome composition [14, 28]. Evidence of host genome influence on microbiome composition has been reported in humans by Goodrich et al. [29], and studies of the pig gut microbiome have reported non-zero heritability estimates of certain microbial taxa [30]. A genome-wide association study has also recently been conducted to explore potential links between single-nucleotide polymorphisms within the pig genome, and the abundance of taxa was shown to be significantly associated with growth and fatness parameters [31]. A significant association between the pig genome and the relative abundance of six relevant genera was also found by Crespo-Piazuelo et al. [28].

Landrace pigs had a more diverse gut microbiome compared with the other two breeds, in agreement with Pajarillo et al. [21]. Some researchers have suggested that very low diversity in a microbiome is a good predictor of poor health status [32, 33]. Landrace pigs were also characterized by higher feed efficiency compared to DR and LW, which is consistent with findings reported by Quan et al. [34] who suggested a higher Shannon index in high feed efficiency crossbred pigs.

The gut microbiome composition of DR pigs was significantly different from that of LR and LW animals, while the LR and LW microbiomes were more similar. At the genus level, *Catenibacterium*, *Clostridium*, and *Turicibacter* significantly influenced the separation between sire and maternal lines across the time points considered. The association between the genus *Catenibacterium* and DR compared with LR and LW was also previously reported by Pajarillo et al. [21]. In mice, it has been demonstrated that prevalent bacterial genera with a higher abundance in the same breed usually have a phylogenetic or functional association with their host [35]. Comparing the two maternal lines, LR pigs had a significantly higher proportion of *Bacteroides* than LW pigs across time points, instead of *Lactobacillus* as reported by Pajarillo et al. [21]. Large White had higher ADFI and FCR compared to LR. It is known in pigs that the type and quantity of feed consumed can affect both host biology and the relative abundance of different bacterial species in the gastrointestinal tract. For example, Frese et al. [36] indicated that the diet composition can widely modify the gut microbiome composition (e.g., increase of *Prevotella*), which reflected the different functional capacities of the microbial community in different time points.

A variation across time point was observed with an increase in abundance of some taxa associated with *Firmicutes* and *Bacteroidetes* in the gut of DR pigs compared to LR and LW. A significant change in the ratio of *Firmicutes* to *Bacteroidetes* in the gut of developing pigs over time and after cohabitation was observed [37, 38]. Changes in the abundance of *Firmicutes* and *Bacteroidetes* have been associated with a modification in the carcass fat deposition [39]. *Bacteroidetes* had a negative correlation with fat mass and had the ability to utilize various sugar derivatives from vegetables in pigs [40].

At the genus level, the microbiome shift over time was mainly related to *Oscillibacter*, *Prevotella*, *Campylobacter*, and *Treponema.* The relative abundance of *Oscillibacter* and *Prevotella* increased as the pigs aged in DR compared to LR and LW. The proportion of *Campylobacter* increased over time in LR compared to LW, while *Treponema* decreased. An increase in the average proportion of *Prevotella* in the 15 weeks after weaning was also observed by Lu et al. [9]. Regarding a possible biological function of these genera in the gastrointestinal tract, *Oscillibacter* was reported as a probiotic and producer of anti-inflammatory metabolites [41], while *Prevotella* is known to be involved in the degradation of both plant-based dietary polysaccharides [42]. A higher relative amount of *Prevotella* was also reported in both high and low feed efficiency Landrace pigs by Tan et al. [19]. Lastly, taxa classified as *Treponema* were the main driver of the enterotype-like group within the pig gut microbiota associated with growth traits [43], which was involved in cellulose and lignin degradation [16].

It is expected that host genetics has the potential to meaningfully influence the gut microbiota [9, 12, 35], and consequently feed efficiency, by favoring or disfavoring microbes that significantly contribute to nutrient digestion and energy harvest. Therefore, the gut microbiome composition could be associated with intestinal morphology and physiology that can impact the production traits such as growth and feed intake [44].

Characterizing the relationship between gut microbial composition and feed efficiency revealed a positive association between four genera (*Lactobacillus*, *Blautia*, *Dorea*, and *Eubacterium*) and feed efficiency. In agreement with our results, Verschuren et al. [45] found a positive effect of *Lactobacillus* on the feed efficiency of three-way cross pigs. Wang et al. [46] reported an effect of *Lactobacillus* on growth and fat deposition in broiler chickens. Some genera of the family *Lactobacillaceae* were involved in the production of antimicrobial bacteriocins, which are related to gut microbiome composition [47]. A species of the genus *Lactobacillus* with the ability to promote intestinal metabolism was identified in a cluster of highly feed-efficient DR and LR pigs [14, 19]. According to this study, Yang et al. [14] and Quan et al. [34] reported positive associations between taxa classified as *Ruminococcaceae* and feed efficiency in DR and crossbred pigs. Interestingly, taxa belonging to *Ruminococcaceae* are able to produce short-chain fatty acids fermenting dietary polymers, such as polysaccharides, that are not degradable by the host [48]. Fermentation products may influence several aspects of the gastrointestinal tract, such as transit time and nutrient digestion [49]. Moreover, previous studies in swine demonstrated that an increase in the production of short-chain fatty acids could improve the absorptive capacity of the intestine, promoting the growth of beneficial bacteria [49], thereby increasing feed efficiency [14, 34].

## Conclusions

Here, an effect of breeds characterized by different feed efficiency on gut microbiome was discovered. Microbiome differences between breeds were found mainly associated with the genera *Catenibacterium*, *Clostridium*, and *Bacteroides*. These results suggest that host genetics has an essential effect on the structure and composition of the pig gut microbiome. Association analyses between the gut microbiome and feed efficiency revealed a positive association between *Blautia*, *Dorea*, *Eubacterium*, *Lactobacillus*, and feed efficiency in pigs. We provide evidence that the gut microbiome was correlated with feed efficiency and fatness traits, which might be relevant to understand how the intestinal microbial community influences the host production traits. Therefore, these results suggest that the intestinal microbial composition can provide important knowledge in order to improve the feed efficiency of pigs in pork industry.

## Methods

### Experimental design and sample collection

Animal use approval was not needed for this study because the data analyzed were from an existing database provided by Smithfield Premium Genetics (Rose Hill, NC, USA). Data points from Duroc (DR) (*n* = 190), Landrace (LR) (*n* = 221), and Large White (LW) (*n* = 204) boars were used. The animals were the progeny of 27, 27, and 44 sires crossed with 119, 153, and 158 dams for DR, LR, and LW, respectively. The growth trial ran concurrently for the three lines from May to December 2017. During the growth trial, all pigs were provided the same pelleted feed and received standard vaccinations and medications on a nucleus farm composed of 8 rooms (Supplementary Table S4 and S5). In this period, animals were kept in single-breed groups with an average count of 11.3 ± 1.3 animals per group (8 pens/room). Each group had access to one single-space Feed Intake Recording Equipment (FIRE) feeder (Osborne Industries, Inc., Osborne, KS, USA). Each FIRE feeder was equipped with a weighing scale (ACCU-ARM Weigh Race; Osborne Industries, Inc., Osborne, KS, USA) to record the body weight of a pig accessing the feeder (Supplementary Figure S2). In addition, a pig’s identifier and animal consumption were recorded every time a pig visits the feeder. Average daily feed intake (ADFI) was calculated as the average amount of feed consumed daily during the growth trail. Average daily gain (ADG) for each tested boar was measured as the ratio between body weight and age across the testing period (Supplementary Figure S3). One estimate of residual feed intake (RF1) was calculated as the residual from a regression model of ADFI on ADG. A second estimate of residual feed intake (RF2) was calculated as for RF1 but also included a correction with respect to body weight as reported by [5]. The average feed conversion ratio (FCR) was calculated as the ratio between ADFI and ADG. At the end of the growth trial, ultrasound backfat, loin eye area, and intramuscular fat (IMF) were measured. Ultrasound images of all animals were recorded with an Aloka 500 ultrasound machine (Corometrics Medical Systems, Wallingford, CT, USA) and analyzed for IMF using the Swine Image Analysis Software (Designer Genes Technologies Inc., Harrison, AR, USA). Descriptive statistics of all the traits are presented in Table 1. Fecal samples were collected by swabbing the rectum of each animal at 73.2 ± 2.9 days, 123.4 ± 3.6 days, and 158.5 ± 4.4 days after birth and were subjected to microbiome sequencing (Supplementary Figure S4). A schematic illustration depicting the experimental design and sample collection is reported in Supplementary Figure S5.

### DNA extraction and 16S rRNA gene amplicon sequencing of fecal

DNA was extracted from each rectal swab using the method reported in detail by Lu et al. [9]. Phased, bi-directional amplification of the V4 region (515–806) of the 16S rRNA gene was employed to generate indexed libraries for Illumina sequencing in the manner previously described [9]. All sequencing was performed at the DNA Sequencing Innovation Lab at the Center for Genome Sciences and Systems Biology at Washington University in St. Louis (USA). The raw sequence data from the Illumina platform were converted into read files using MiSeq Reporter. Pairs of V4 16S rRNA gene sequences were first merged into a single sequence using FLASH v1.2.11 [50] with a required overlap of at least 100 and no more than 250 base pairs in order to provide a confident overlap. Sequences were oriented in the forward direction, and any primer sequences were trimmed; during primer matching, up to 1 mismatch was allowed. Sequences were imported into Quantitative Insights Into Microbial Ecology (QIIME2 version 2017.12, https://qiime2.org/) for demultiplexing and the construction of an amplicon sequence variant (ASV) feature table using the Divisive Amplicon Denoising Algorithm 2 (DADA2) [51] with default settings and no truncation or length filtering (--p-trunc-len 0). Features present in only 1 sample were removed from the table. Finally, the Ribosomal Database Project (RDP) classifier (v2.4) was retrained in the manner previously described [52] and used to predict taxonomic assignments for each ASV sequence using a confidence cutoff of 0.8. Results were then exported for further analysis in the R environment [53].

### Data editing

Feeding efficiency traits and body weight were obtained from the FIRE system records. To obtain an accurate prediction of individual feed intake and body weight, data editing was required [5]. In this study, feed intake records were edited on the basis of the procedure proposed by [5, 54], while body weights were edited following the procedure reported by [26, 55]. We fit a robust regression model using the “mass” [56] package in R [53], with age and squared age as covariates. Each data point was assigned a weight (from 0 to 1) to minimize the influence of extreme values. Data with weights of less than 0.5 units were treated as outliers and removed before any statistical analysis [55]. The predicted body weight from robust regression was used to calculate ADG for each boar. Average daily gain values that were outside the mean plus or minus three standard deviations were considered outliers and removed. The final data set comprised of 336,959 data points. We focused our analysis using ASV with a total count ≥ 1000 across all samples within a time point. In total, 765, 729, and 820 ASV met these criteria in the final data set for 73, 123, and 158 days, respectively.

### Statistical analyses

#### Phenotypic differences

To investigate the impact of breed and to control the environmental factors on performance, growth and efficiency traits were analyzed using the following model with the MIXED Procedure in SAS 9.4 [57].

1 $$ {y}_{ijklm}=\mu +{Br}_i+{Ro}_j+ Si{(Br)}_{k:i}+ Pe{(Ro)}_{l:j}+{e}_{ijklm}, $$

where *y*_*ijklm*_ was the overall mean of the observed trait (ADFI, ADG, RF1, RF2, FCR, backfat, loin depth, and IMF); *μ* was the overall intercept of the model; *Br*_*i*_ was the fixed effect of the *i*th Breed (*i* = 1 to 3); *Ro*_*j*_ was the fixed effect of the *j*th Room (*j* = 1 to 8); *Si(Br)*_*k:i*_ was the random effect of the *k*th sire (*k* = 1 to 100) within Breed which was assumed to be N(0,**I**${\sigma}_{Si}^2$); *Pe(Ro)*_*l:j*_ was the random effect of the *l*th pen (*l* = 1 to 64) within *Ro* which was assumed to be N(0,**I**${\sigma}_{Pe}^2$); and *e*_*ijklm*_ was the residual error which was assumed to be N(0,**I**${\sigma}_e^2$), where σ_e_^2^ was the residual variance.

Moreover, to assess the relationships between different breeds, a linear discriminant analysis (LDA) was performed using the “lda” function in the “mass” package [56] in R and overall mean of feed efficiency and fatness traits as variables.

#### Microbiome composition differences across breeds

The ASV for 73, 123, and 158 days were analyzed using a negative binomial model with the “edgeR” [58] package in R, including the fixed effects described in the model (1). Orthogonal contrasts were fitted as reported above to obtain ASV significance among breeds. The Benjamini–Hochberg method was used for multiple-testing correction [59]. A false discovery rate of 5% was used to declare whether or not an ASV was significantly different between breeds. Jensen-Shannon Divergence [60] was calculated for each breed at the three separate time points according to the relative abundance of each genus in each sample using the “dist.JSD” function from the phyloseq package in R [61, 62] to identify enterotypes among the samples. Based on the obtained distance matrix, the samples at each time point were clustered via partitioning around medoids by using the “pam” function in the R package “cluster” [63]. The optimal number of clusters was chosen by maximizing the Calinski–Harabasz index [64], using the “index.G1” function in the R package “clusterSim” [65], and the Silhouette index [66], using the “silhouette” function in the R package “cluster” [63]. To identify genera that had a significant effect on the division of the enterotypes at each time point, the LDA Effect Size based on the non-parametric Kruskal–Wallis sum-rank test was performed [67] with an alpha value of 0.05 for the factorial Kruskal–Wallis test among classes and a threshold of 2 on the logarithmic LDA score for discriminative features using the galaxy/hutlab website (http://huttenhower.sph.harvard.edu/galaxy/).

#### ASV–phenotype association

We investigated the effect of ASV at 73, 123, and 158 days on performance traits and residual feed intake using the following model with the MIXED Procedure in SAS 9.4 [57]:

2 $$ {y}_{ijklmn}=\mu +{Br}_i+{Ro}_j+{As}_k+ As\times {Br}_{ik}+ Si{(Br)}_{l:i}+ Pe{(Ro)}_{m:j}+{e}_{ijklmn}, $$

where *y*_*ijklmn*_ was the phenotype within each time points (ADFI, ADG, RF1, RF2, FCR, backfat, loin depth, and IMF); *μ* was the overall intercept of the model; *Br*_*i*_ and *Ro*_*j*_ were the fixed effects described in the model (1); *As*_*k*_ was the covariate of the *k*th ASV; *As × Br*_*ik*_ was the fixed effect of the ik*th As × Br*_*ik*_ interaction; *Si(Br)*_*l:i*_, *Pe(Ro)*_*m:j*_, and *e*_*ijklmn*_ were as in the model (1). Orthogonal contrasts among breeds were fitted as reported above. An FDR of 5% was used to declare whether or not an ASV was significantly associated with traits and/or statistically different between breeds. Spearman correlations were used to correlate feed efficiency, fatness traits, and ASVs with a genus assignment using the “Hmisc” (https://cran.r-project.org/web/packages/Hmisc/Hmisc.pdf) package in R.

## Supplementary information

**Additional file 1.** Descriptive statistics of feed efficiency and fatness traits of pigs.

**Additional file 2.** Relative abundance of amplicon sequence variant (ASV) at genus level in the gut microbiome of pigs.

**Additional file 3.** Spearman correlations between gut microbiome, feed efficiency, and fatness traits of pigs.

**Additional file 4: Supplementary Table S1.** Differences between the gut microbiome of three breeds of pigs.

**Additional file 5: Supplementary Table S2.** Results of ASV effect on feed efficiency and fatness traits of three purebred lines across three time points of the growth trial.

**Additional file 6: Supplementary Table S3.** Results of the interaction between ASV and breed effect on feed efficiency and fatness traits of three purebred lines across three time points of the growth trial.

**Additional file 7: Supplementary Table S4.** Composition of the diets fed to pigs in this study.

**Additional file 8: Supplementary Table S5.** Medical treatments administered to pigs in this study.

**Additional file 9: Supplementary Figure S1.** Calinski-Harabasz indexes (CH) for number of potential clusters of samples at 73 days (T1), 123 days (T2), and 158 days (T3) for Duroc (red), Landrace (blue), and Large White (green). The highest CH value at each time point indicates optimal number of cluster/enterotypes.

**Additional file 10: Supplementary Figure S2.** Variation of body weight of Duroc (DR), Landrace (LR) and Large White (LW) during the feeding trial.

**Additional file 11: Supplementary Figure S3.** Average daily gain of Duroc (DR), Landrace (LR) and Large White (LW) during the feeding trial.

**Additional file 12: Supplementary Figure S4.** Average age of Duroc (DR), Landrace (LR) and Large White (LW) during the feeding trial.

**Additional file 13: Supplementary Figure S5.** Illustration of the experimental design.

## Data Availability

The phenotypes generated and/or analyzed during the current study are not publicly available due to third-party ownership but are available from the corresponding authors upon reasonable request. 16S rRNA gene sequences will be updated to a public repository upon acceptance of the manuscript for publication.

## Abbreviations

ASV: Amplicon sequence variant
ADG: Average daily gain
ADFI: Average daily feed intake
DR: Duroc
FCR: Feed conversion ratio
FIRE: Feed Intake Recording Equipment
FDR: False discovery rate
IMF: Intramuscular fat
LR: Landrace
LDA: Linear discriminant analysis
LW: Large White
RF1: Residual feed intake 1 (calculated by regressing ADFI on ADG)
RF2: Residuals feed intake 2 (calculated by regressing ADFI on ADG and body weight)
