# Supplementary material for: Gut microbiome composition differences among breeds impact feed efficiency in swine
Source: Microbiome. 2020 Jul 22;8:110. doi: 10.1186/s40168-020-00888-9 (PMC7376719; doi:10.1186/s40168-020-00888-9)
Supplement: Supplementary file 2 — Additional file 1. Descriptive statistics of feed efficiency and fatness traits of pigs. [file 40168_2020_888_MOESM1_ESM.pdf]

Additional file 1. Descriptive statistics for efficiency and fatness traits.

Item	Duroc		Landrace		Large White	
	Mean	SD	Mean	SD	Mean	SD
Animals, n	190		221		204	
Efficiency traits:						
ADFI, g/d	2,154.1	326.1	2,203.2	360.3	2,384.5	354.2
ADG, g/d	615.2	68.2	628.3	75.4	650.9	82.1
RF1, g	0.00	341.2	0.00	287.0	0.00	287.1
RF2, g	0.00	307.8	0.00	279.5	0.00	263.8
FCR	3.53	0.54	3.51	0.58	3.67	0.45
Fatness traits:						
Back fat, mm	10.12	2.63	11.69	2.95	14.04	3.20
Loin depth, mm	49.08	6.63	48.52	6.73	47.07	6.59
IMF, %	2.09	0.89	1.84	0.75	1.93	0.79

ADFI = average daily feed intake; ADG = average daily gain; RF1 = residuals calculated regressing ADFI on ADG; RF2 = residuals calculated regressing ADFI on ADG and body weight; FCR = average feed conversion ratio calculated as the ratio between ADFI and ADG; IMF = intramuscular fat.
