# Supplementary figures and images for: Gut microbiome composition differences among breeds impact feed efficiency in swine
Source: Microbiome. 2020 Jul 22;8:110. doi: 10.1186/s40168-020-00888-9 (PMC7376719; doi:10.1186/s40168-020-00888-9)

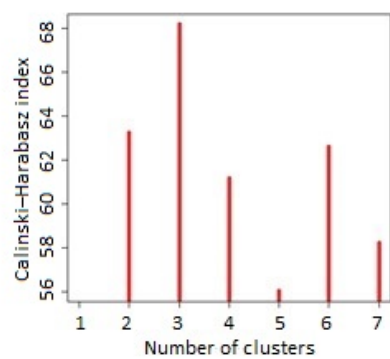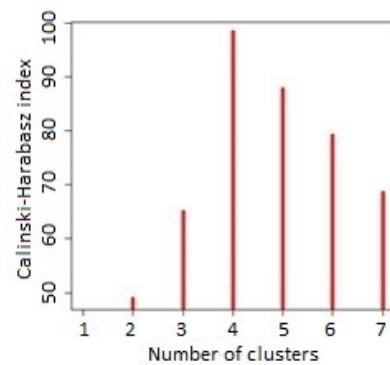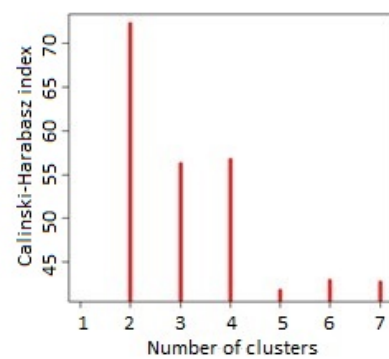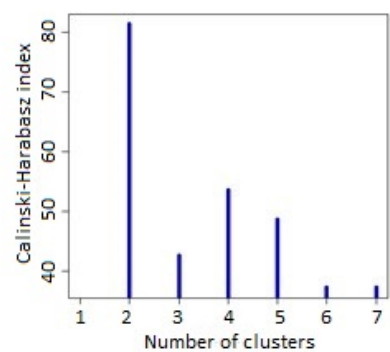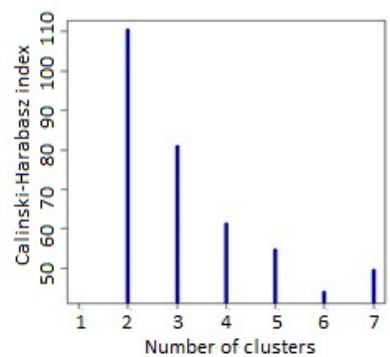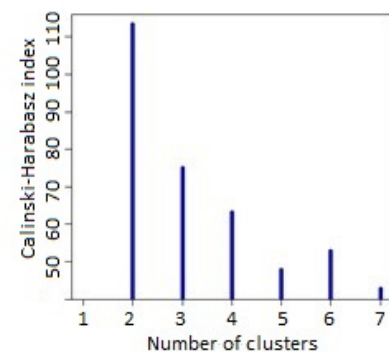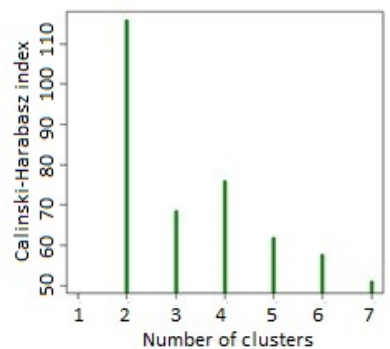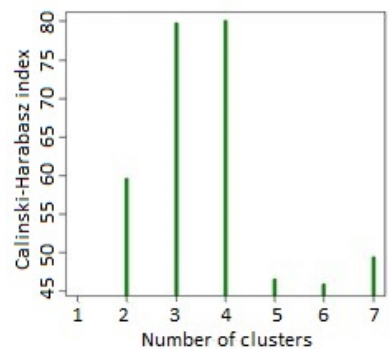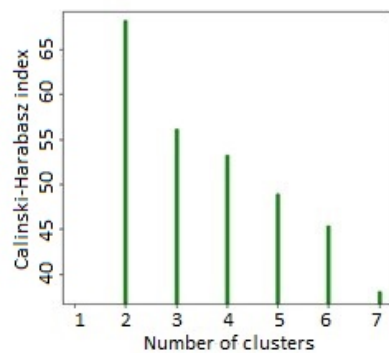

Supplement: Supplementary file 10 — Additional file 9: Supplementary Figure S1. Calinski-Harabasz indexes (CH) for number of potential clusters of samples at 73 days (T1), 123 days (T2), and 158 days (T3) for Duroc (red), Landrace (blue), and Large White (green). The highest CH value at each time point indicates optimal number of cluster/enterotypes. [file 40168_2020_888_MOESM9_ESM.pdf]

## Body weight

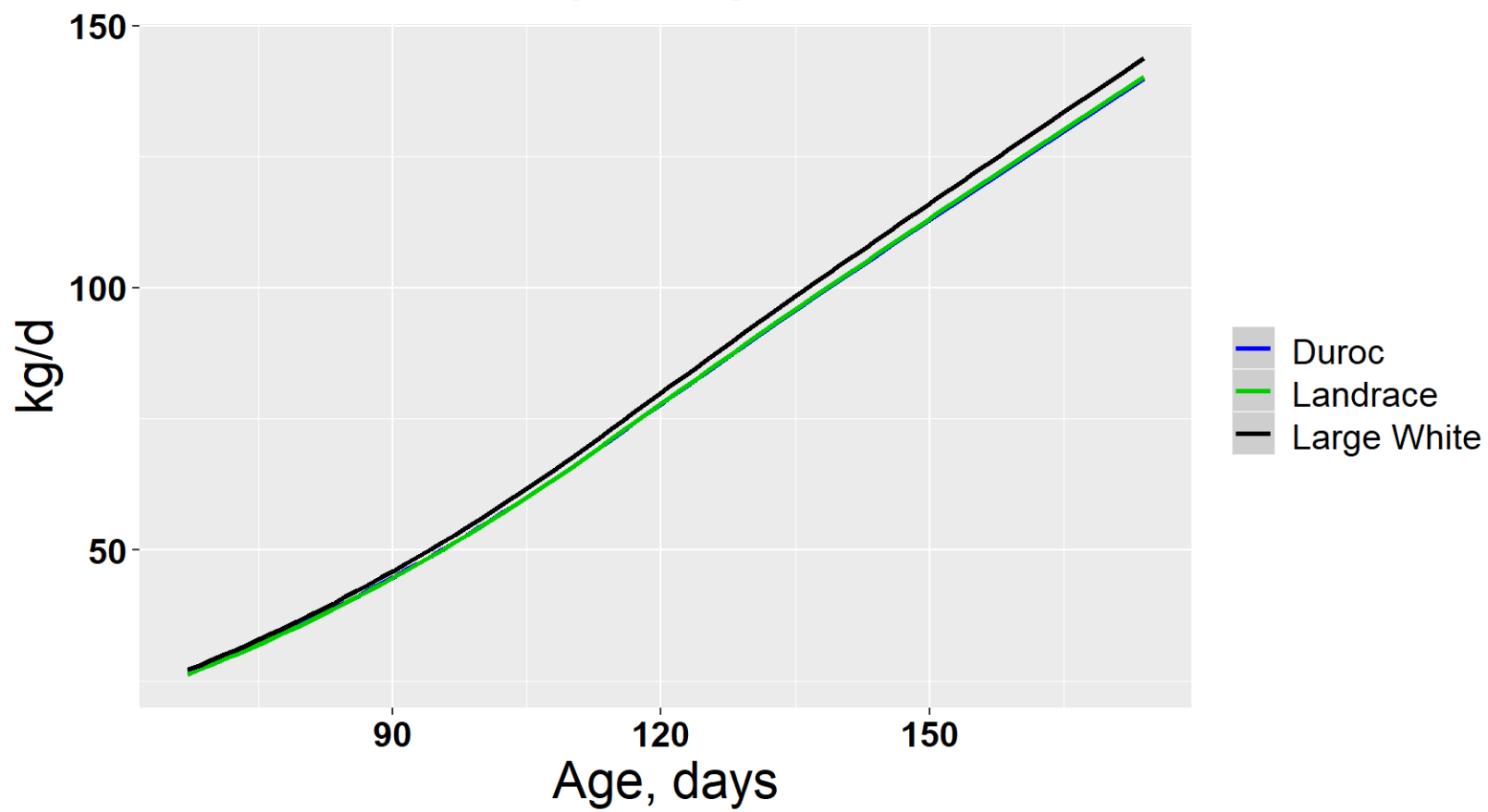

Supplement: Supplementary file 11 — Additional file 10: Supplementary Figure S2. Variation of body weight of Duroc (DR), Landrace (LR) and Large White (LW) during the feeding trial. [file 40168_2020_888_MOESM10_ESM.pdf]

## Average Daily Gain

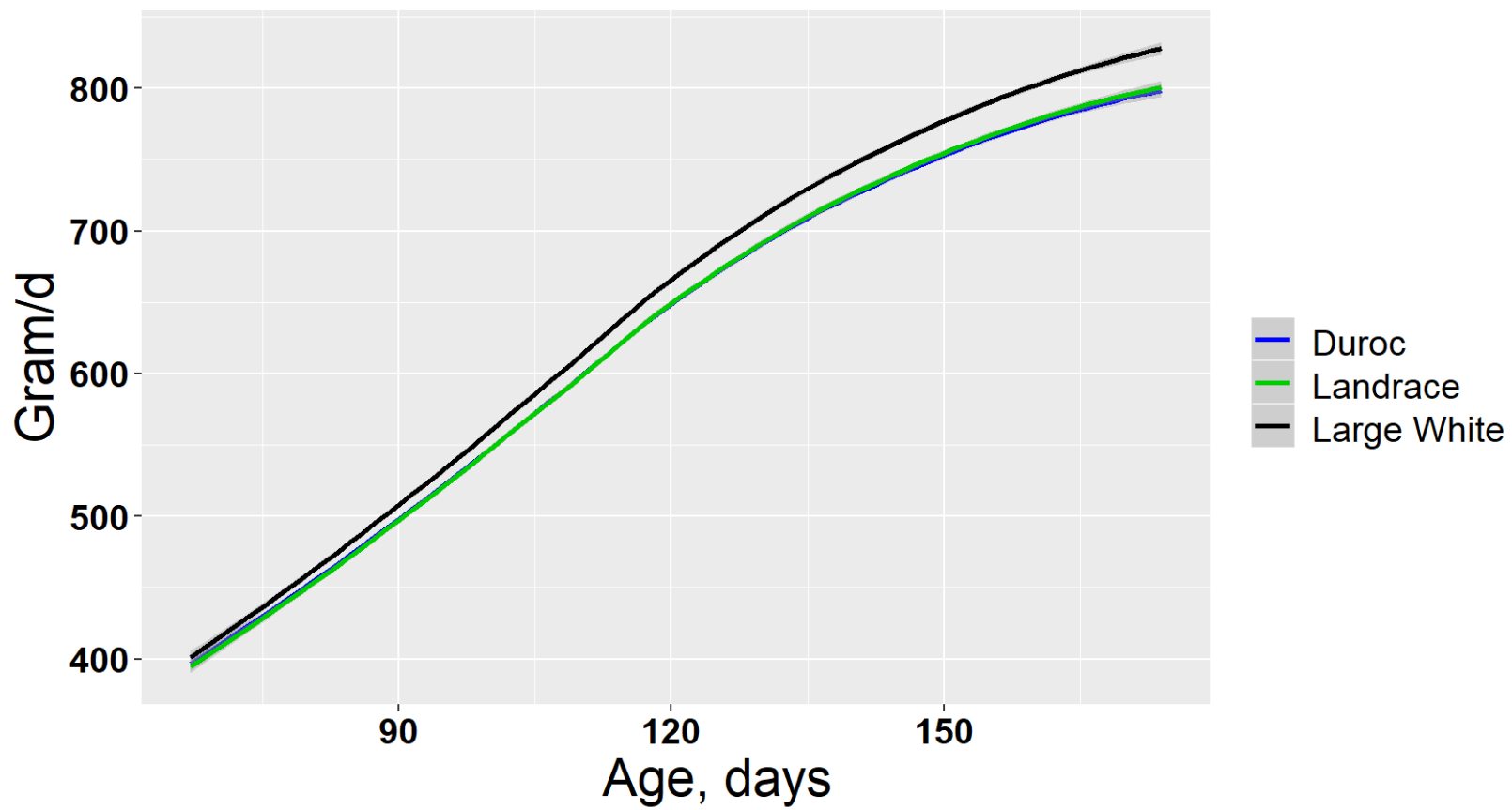

Supplement: Supplementary file 12 — Additional file 11: Supplementary Figure S3. Average daily gain of Duroc (DR), Landrace (LR) and Large White (LW) during the feeding trial. [file 40168_2020_888_MOESM11_ESM.pdf]

# Animal age

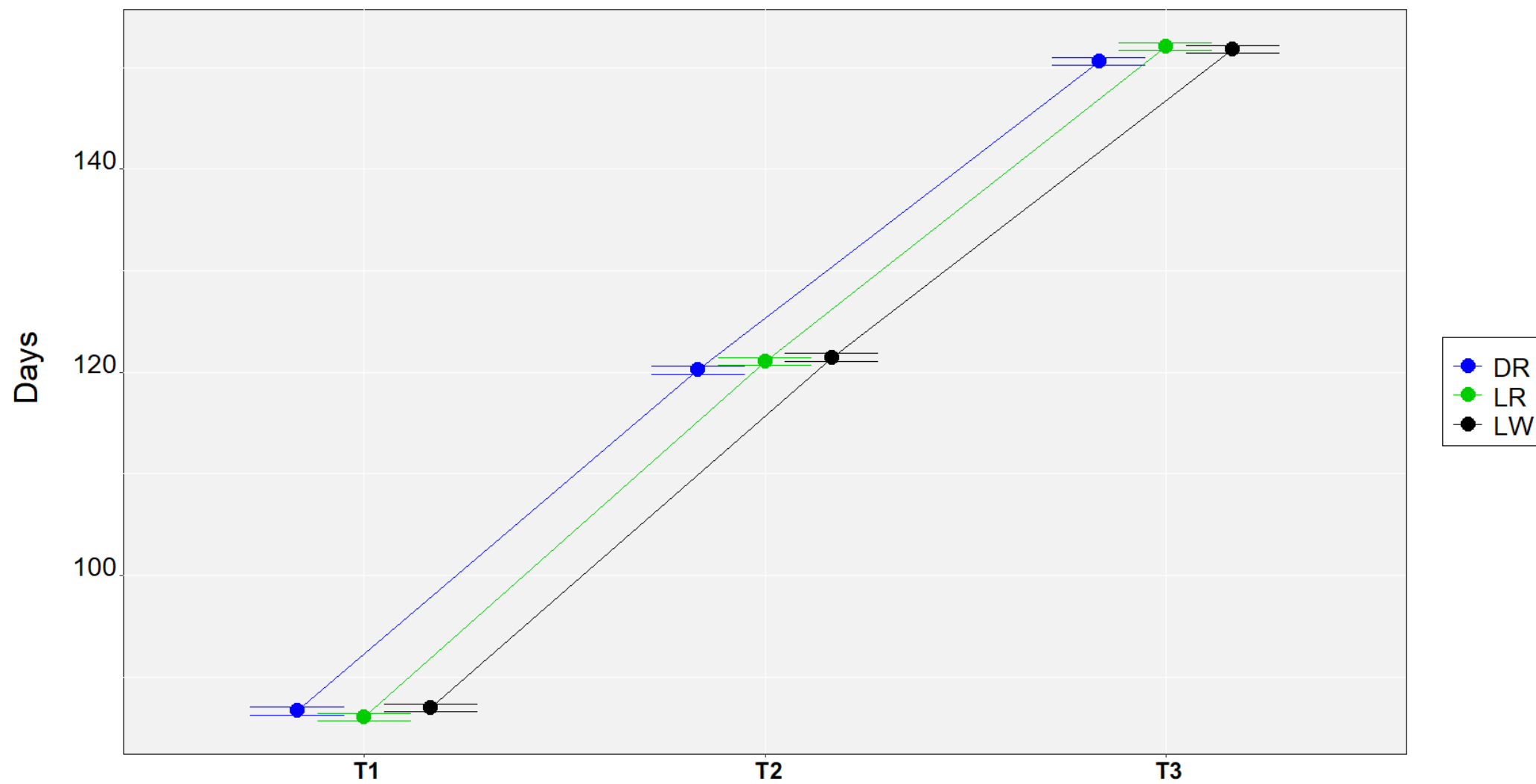

Supplement: Supplementary file 13 — Additional file 12: Supplementary Figure S4. Average age of Duroc (DR), Landrace (LR) and Large White (LW) during the feeding trial. [file 40168_2020_888_MOESM12_ESM.pdf]

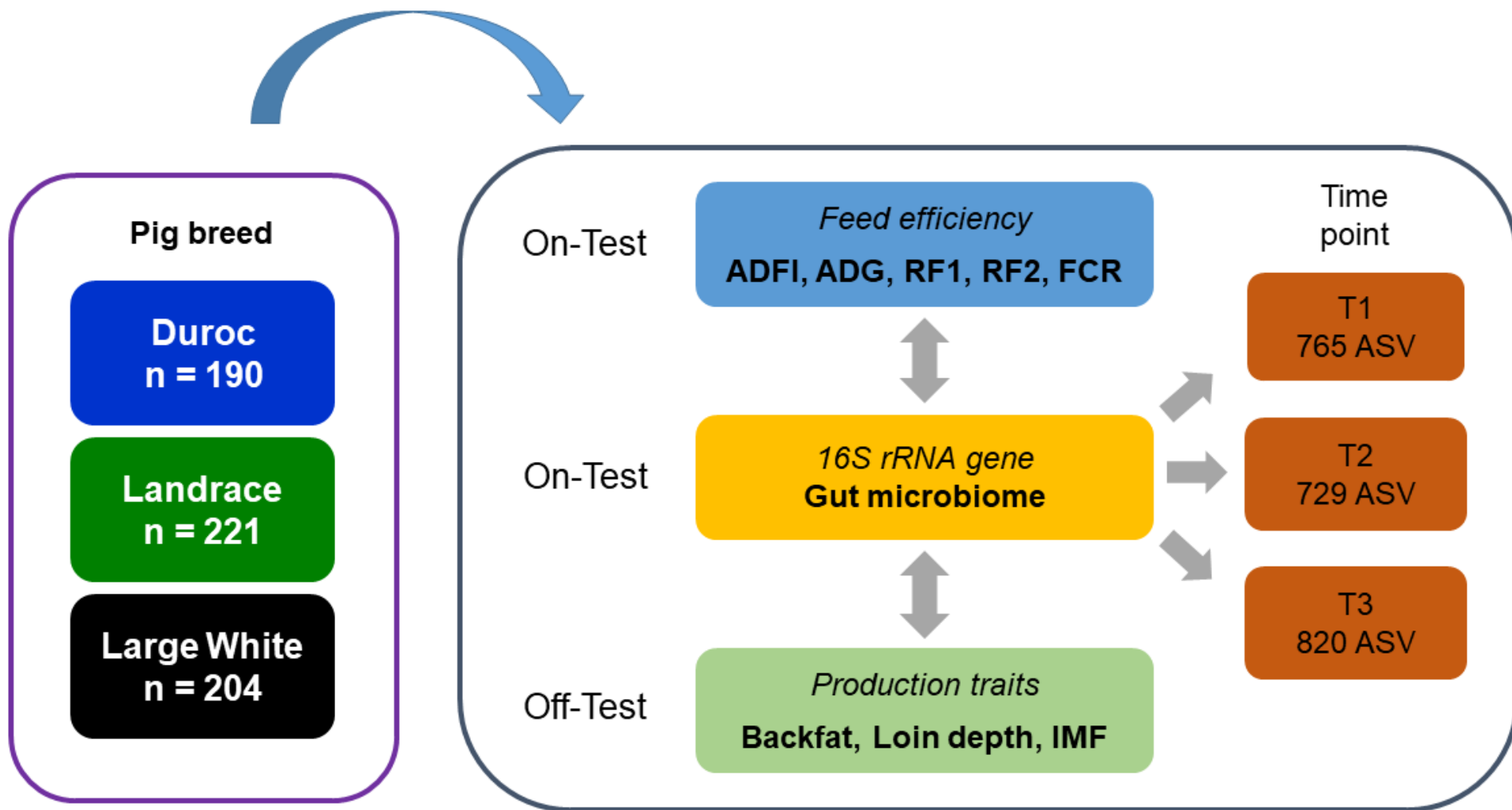

Supplement: Supplementary file 14 — Additional file 13: Supplementary Figure S5. Illustration of the experimental design. [file 40168_2020_888_MOESM13_ESM.pdf]
